# Presentation, management, and outcomes of orbital infections in patients with cancer

**DOI:** 10.3389/fopht.2025.1676014

**Published:** 2025-11-17

**Authors:** Ava Niknahad, Alison B. Gibbons, Aishah Ahmed, Heya Lee, Setu Mehta, Samir Al-Ali, Meron Haile, Anjana Srikumar, Rachel Stemme, Akanksha Suresh, Justin C. Zhong, Fasika Woreta, Emily Li, Fatemeh Rajaii

**Affiliations:** 1 Wilmer Eye Institute, Johns Hopkins University, Baltimore, MD, United States; 2 Johns Hopkins University, Baltimore, MD, United States

**Keywords:** orbital infection, orbital cellulitis, preseptal cellulitis, cancer, immunosuppression

## Abstract

**Purpose:**

This study aimed to characterize the presentation, treatment, and outcomes of preseptal and orbital cellulitis in patients with and without cancer, to help guide management of these infections.

**Methods:**

A retrospective cohort study was conducted at a tertiary care center to identify adults who presented from 2007 to 2022 with orbital and preseptal cellulitis. Patients with cancer were defined as patients actively receiving chemotherapy or in remission but on immunosuppressants at the time of their orbital infection care. Their demographics, presentation, management, and follow-up characteristics were recorded. Management variables included data on antibiotics, antifungals, and systemic steroids administered.

**Results:**

Of the 183 patients presenting with orbital cellulitis, 15 (8.2%) had active cancer while of the 130 patients with preseptal cellulitis, 8 (6.2%) had cancer (*p* = 0.661). Patients with cancer who were found to have preseptal cellulitis were more likely to have head and neck cancer than those who had orbital cellulitis (50.0 versus 0%, *p* = 0.008). In the orbital cellulitis cohort, the management of patients with and without cancer differed in antifungal and corticosteroid therapy. Compared to patients without cancer, patients with cancer received a higher antifungal rate (46.7% versus 10.1%, *p* = 0.001) and were started on steroids a median of 2 days later (3.0 versus 1.0 day after admission date, *p* = 0.007). Both cohorts had similar readmission rates (20.0% in cancer and 11.3% in non-cancer cohort, *p* = 0.397) and eye exam findings at follow-up. Patients with and without cancer in the preseptal cellulitis cohorts were similar in all characteristics assessed.

**Conclusions:**

Patients with cancer presenting with orbital cellulitis have a higher rate of antifungal administration and are started on systemic steroids later compared to patients without cancer, although they have similar eye exam outcomes and readmission rates. Patients with cancer are not at an increased risk of preseptal cellulitis and are managed similarly to patients without cancer.

## Introduction

Preseptal cellulitis, also known as periorbital cellulitis, is an infection of the orbital region anterior to the orbital septum (1). Orbital cellulitis is an infection of the orbital region posterior to the orbital septum, encompassing the eyeball and deeper tissue (1). Both conditions may present similarly with periorbital redness, swelling, fever, or pain. However, additional features of orbital cellulitis such as proptosis, afferent pupillary defect, ophthalmoplegia, or orbital pain accompanying eye movement help clinicians differentiate preseptal from orbital cellulitis (1, 2). While preseptal cellulitis is often treated with oral antibiotics, orbital cellulitis requires hospital admission, treatment with intravenous antibiotics, and in some cases surgical intervention (1–3). If not treated appropriately, orbital cellulitis can lead to complications such as vision loss, intracranial extension, or death (1, 3–5). For example, if the infection leads to an intracranial abscess, the mortality rate increases from 1-2% to 40% (6). This is in contrast to preseptal cellulitis which typically resolves with outpatient treatment on oral antibiotics, highlighting the urgency and importance of distinguishing preseptal from orbital cellulitis for proper management (7).

Appropriate management of preseptal cellulitis and orbital cellulitis is especially critical in immunocompromised patients due to the potential for more severe bacterial or fungal infections. Patients with cancer are part of such a group due to treatment-induced immune suppression (8). For example, in a study of 160 patients with colorectal cancer, acute bacterial infection during or after the end of treatment was found to be a significant predictor of poor survival (hazard ratio 2.62) (9). In another study on hematopoietic cell transplant recipients and patients with acute myeloid leukemia and myelodysplastic syndrome, overall mortality rate from invasive fungal infections was found to be 47% (10). If cancer involves the immune system itself, the lack of properly proliferating and developing immune cells pose an additional risk for a weakened immune system, thus acquiring an infection (8). However, given the incidence of orbital cellulitis in adults at a rate of 0.1 per 100,000 adults (6), there is little data in the literature characterizing adult patients with cancer who present with preseptal or orbital cellulitis (11). As such, the goal of our study is to define and compare the clinical characteristics and management course of preseptal and orbital cellulitis in patients with cancer compared to patients without cancer presenting to a tertiary care center. By doing so, this study aims to provide clinicians with a summary of presenting characteristics and management of orbital cellulitis in patients with cancer and elucidate the outcomes in this population.

## Methods

This retrospective cohort study was approved by the JHH Institutional Review Board (IRB00132759). Collection and evaluation of protected patient health information were in compliance with the Health Insurance Portability and Accountability Act and adhered to the ethical principles outlined in the Declaration of Helsinki. Our inclusion criteria included adults (18 years or older) who presented to the Johns Hopkins Health system from 2007 to 2022 for diagnosis and treatment of preseptal or orbital cellulitis. Patients were diagnosed with preseptal or orbital cellulitis clinically, with imaging acquired when needed to support clinical diagnosis. Exclusion criteria included patients with a final diagnosis of preseptal cellulitis but exam findings of rapid afferent pupillary defect (RAPD) or extraocular movement (EOM) restriction, without a documentation of orbital cellulitis as a diagnosis, given that RAPD and EOM restriction are signs of orbital cellulitis rather than preseptal cellulitis. Patients with diagnosis of preseptal cellulitis but no antibiotic treatment and diagnosis of orbital cellulitis but no hospital admission for intravenous antibiotics were excluded as their treatments differed from the standard of treatment needed for these conditions.

Patients were separated into preseptal and orbital cellulitis cohorts, and within each cohort, they were divided into patients with and without a previous cancer diagnosis. Inclusion criteria of the cancer cohort included patients who were actively receiving chemotherapy or were on immunosuppressants while on remission at the time of their orbital infection diagnosis and treatment.

For each of the cohorts, patient demographics, presentation, management, and follow-up characteristics were recorded. Presentation characteristics included location of first presentation, hospital admission, laterality and etiology of cellulitis, and eye exam at presentation. Follow-up characteristics included time from presentation to follow-up, eye exam at that time, and readmission rates. When available, the eye exam included best corrected visual acuity (BCVA), presence of RAPD, intraocular pressure (IOP), Ishihara color tests, and EOM restriction. Management characteristics included length of stay at the hospital if admitted, and whether surgical management or medical management using antibiotics, steroids, and antifungals were used. Duration of intravenous (IV) antibiotics, dosing of steroid in prednisone (mg) equivalent, and duration of antifungals were recorded. At this tertiary care center, steroids are often initiated when patients fail to respond to antibiotics. Antifungals are started based on the overall clinical risk of fungal infection or in non-healing immunocompromised patients.

The cohorts were analyzed using STATA 17.0 (StataCorp LLC, College Station, TX). Nominal and continuous outcomes were respectively compared using Fisher’s exact and Mann-Whitney U tests, comparing either preseptal and orbital cellulitis cohorts or patients with and without cancer within each cellulitis cohort. Statistical significance was determined by a *p-value* < 0.05.

## Results

### Demographics of orbital cellulitis and preseptal cellulitis patients

Of the 313 patients that met inclusion criteria, 183 had orbital cellulitis and 130 had preseptal cellulitis. Fifteen (8.2%) of the patients with orbital cellulitis had a concomitant cancer diagnosis as compared to 8 (6.2%) patients with preseptal cellulitis (*p* = 0.661, **Table 1**). Patients with cancer who were found to have preseptal cellulitis were more likely to have head and neck cancer than those who had orbital cellulitis (50.0 versus 0%, *p* = 0.008). In both preseptal and orbital cellulitis cohorts, patients with cancer were approximately 10 years older than patients without cancer although this difference was not significant (respectively, *p* = 0.117 and *p* = 0.105, **Table 2**). Patients with and without cancer had similar demographics in both cellulitis cohorts (**Table 2**).

**Table 1:** Patients with cancer characteristics by orbital infections.

	All patients (*N* = 313)	Preseptal cellulitis (*N* = 130)	Orbital cellulitis (*N* = 183)	*P-value*
Patients with cancer	23 (7.4%)	8 (6.2%)	15 (8.2%)	0.661
Treatment status				
Actively receiving chemotherapy	19 (82.6%)	5 (62.5%)	14 (93.3%)	0.103
In remission receiving immunosuppression	4 (17.4%)	3 (37.5%)	1 (6.7%)	0.103
Type of cancer				
Hematologic	13 (56.5%)	3 (37.5%)	10 (66.7%)	0.221
Head and Neck	4 (17.4%)	4 (50.0%)	0 (0%)	0.008*
Other solid tumors	6 (26.1%)	1 (12.5%)	5 (33.3%)	0.369

*P-*values were calculated using Fisher’s exact tests for nominal outcomes, comparing the preseptal and orbital cellulitis cohorts. *p-value*<*0.05

**Table 2:** Preseptal and orbital cellulitis demographics.

Preseptal Cellulitis	Patients without Cancer (*N* = 122)	Patients with Cancer (*N* = 8)	*P-value*
Median age at diagnosis, years (IQR)	50.9 (35.4-64.0)	61.0 (53.6-67.5)	0.117
Race			
White Black	75 (61.5%) 36 (29.5%)	4 (50.0%) 4 (50.0%)	0.711 0.250
Asian	2 (1.6%)	0 (0%)	1.000
Other	9 (7.4%)	0 (0%)	1.000
Sex			
Female	76 (62.3%)	6 (75.0%)	0.709
Male	46 (37.7%)	2 (25.0%)	0.709
Orbital Cellulitis	Patients without Cancer (*N* = 168)	Patients with Cancer (*N* = 15)	*P*-value
Median age at diagnosis, years (IQR)	52.1 (35.7-64.9)	61.9 (49.4-72.6)	0.105
Race			
White	97 (57.7%)	9 (60.0%)	1.000
Black	55 (32.7%)	2 (13.3%)	0.152
Asian	3 (1.8%)	1 (6.7%)	0.292
Other	13 (7.7%)	3 (20.0%)	0.130
Sex			
Female	88 (52.4%)	10 (66.7%)	0.419
Male	80 (47.6%)	5 (33.3%)	0.419

IQR, interquartile range; *p-*values were calculated using Fisher’s exact tests for nominal outcomes and Mann-Whitney U tests for continuous outcomes, comparing patients without cancer and with cancer cohorts.

### Preseptal cellulitis cohort: clinical characteristics and management

**Table 3** summarizes the clinical characteristics and hospital course of patients with preseptal cellulitis. Of the patients with preseptal cellulitis, both those with and without a concomitant cancer diagnosis were more likely to present to the emergency room for initial evaluation instead of to the primary care practice or an ophthalmologist. Patients with cancer were admitted at a higher rate (75%) than patients without cancer (56.6%, *p* = 0.466), and their management with antibiotics, steroids, or antifungals were similar.

**Table 3:** Preseptal cellulitis clinical characteristics and hospital course.

	Patients without Cancer (*N* = 122)	Patients with Cancer (*N* = 8)	*P*-value
First presentation location			
Primary care practice Emergency department	12 (9.8%) 104 (85.3%)	1 (12.5%) 6 (75.0%)	0.580 0.356
Ophthalmologist	4 (3.3%)	0 (0%)	1.000
Other	2 (1.6%)	1 (12.5%)	0.175
Hospital admission	69 (56.6%)	6 (75.0%)	0.466
Management			
Antibiotics during admission	64 (92.8%)	5 (83.3%)	0.405
Antibiotics at discharge	122 (100%)	8 (100%)	NA
Steroids during admission	5 (7.3%)	1 (16.7%)	0.405
Day of first steroids administered from admission, (IQR)	2.0 (2.0-2.0)	0 (0-0)	0.667
Antifungals during admission	5 (7.3%)	2 (33.3%)	0.094
Median duration of antifungals, days (IQR)	2.0 (1.0-8.0)	2.5 (2.0-3.0)	0.643

IQR, interquartile range; NA, not applicable; *p-*values were calculated using Fisher’s exact tests for nominal outcomes and Mann-Whitney U tests for continuous outcomes, comparing patients without cancer and with cancer cohorts.

### Orbital cellulitis cohort: clinical characteristics and management

Of patients with orbital cellulitis, both those with and without cancer were most likely to first present to the emergency department (66.7% versus 83.9% respectively, **Table 4**). When a potential etiology was mentioned in the records, sinusitis was the most common etiology in both patients with and without cancer (40.0% versus 36.9% respectively, *p* = 0.788). All patients presenting with orbital cellulitis were admitted to hospital for management, with a median length of admission of 6.0 days for patients with cancer and 4.0 days for patients without cancer (*p* = 0.232).

**Table 4:** Orbital cellulitis clinical characteristics and hospital course.

	Patients without Cancer (*N* = 168)	Patients with Cancer (*N* = 15)	*P*-value
Laterality			
Right	81 (48.2%)	4 (26.7%)	0.175
Left	84 (50.0%)	11 (73.3%)	0.107
Both	3 (1.8%)	0 (0%)	1.000
Etiology			
Sinusitis	62 (36.9%)	6 (40.0%)	0.788
Trauma	26 (15.5%)	1 (6.7%)	0.702
Dental infection	11 (6.7%)	1 (6.7%)	1.000
Other causes	8 (4.8%)	1 (6.7%)	0.545
None mentioned in EMR	61 (36.3%)	6 (40.0%)	0.785
First presentation location			
Primary care physician	4 (2.4%)	0 (0%)	1.000
Emergency department	141 (83.9%)	10 (66.7%)	0.146
Ophthalmologist	12 (7.1%)	3 (20.0%)	0.111
Other	11 (6.6%)	2 (13.3%)	0.289
Hospital admission			
Overall	168 (100%)	15 (100%)	NA
From emergency department	141 (100%)	10 (100%)	NA
Median length of stay, days (IQR)	4.0 (3.0-7.0)	6.0 (4.0-12.0)	0.232

IQR, interquartile range; EMR, electronic medical records; NA, not applicable; *p-*values were calculated using Fisher’s exact tests for nominal outcomes and Mann-Whitney U tests for continuous outcomes, comparing patients without cancer and with cancer cohorts.

Compared with patients without cancer, patients with cancer were significantly less likely to undergo surgery (13.3% versus 43.5%, *p* = 0.028, **Table 5**), and when surgery was performed, it occurred later after presentation (median 14.0 versus 1.0 days, *p* = 0.003). Antibiotic use did not differ between groups, but steroids were initiated significantly later in patients with cancer (median 3.0 versus 1.0 days after presentation, *p* = 0.007). Patients with cancer were also significantly more likely receive antifungal therapy (46.7% versus 10.1%, *p* = 0.001).

**Table 5:** Orbital cellulitis management characteristics.

	Patients without Cancer (*N*=168)	Patients with Cancer (*N* = 15)	*P*-value
Su*rgery:*			
Surgery performed	73 (43.5%)	2 (13.3%)	0.028*
Time to surgery, days (IQR)	1.0 (1.0-2.0)	14.0 (13.0-15.0)	0.003*
Surgery service			
Ophthalmology	33 (45.2%)	2 (100%)	0.740
Otolaryngology	16 (21.9%)	0 (0%)	0.369
Both ophthalmology and otolaryngology	24 (32.9%)	0 (0%)	0.225
*Antibiotics:*			
During admission:			
IV antibiotics	168 (100%)	15 (100%)	NA
Duration of IV antibiotics, days (IQR)	4.0 (3.0-7.0)	4.0 (3.0-8.0)	0.332
At discharge:			
IV antibiotics	17 (10.1%)	0 (0%)	0.367
PO antibiotics	139 (82.7%)	12 (80.0%)	0.729
*Steroids:*			
During admission:			
Number of patients	43 (25.6%)	3 (20.0%)	0.764
Administration start date from admission, day (IQR)	1.0 (1.0-2.0)	3.0 (3.0-15.0)	0.007*
Median total prednisone per weight, mg/kg (IQR)	2.43 (1.71-6.50)	3.95 (0.40-6.30)	0.827
Median total prednisone per weight or day, mg/kg/day (IQR)	0.91 (0.51-2.09)	0.44 (0.40-1.05)	0.369
At discharge	25 (14.9%)	3 (20.0%)	0.706
*Antifungals:*			
During admission:			
Number of patients	17 (10.1%)	7 (46.7%)	0.001*
Median duration of antifungals, days (IQR)	6.0 (1.0-18.0)	2.0 (1.0-4.0)	0.395

IQR, interquartile range; IV, intravenous; NA, not applicable; PO, oral; *p-*values were calculated using Fisher’s exact tests for nominal outcomes and Mann-Whitney U tests for continuous outcomes, comparing patients without cancer and with cancer cohorts.*p-value<0.05

Patients with cancer and without cancer had similar findings on initial exams and follow up visits, without differences in visual acuity, relative afferent pupillary defect, intraocular pressure, Ishihara color test, and extraocular muscle movements (**Table 6**).

**Table 6:** Orbital cellulitis presentation and follow-up eye exams.

	Patients without Cancer (*N* = 168)	Patients with Cancer (*N* = 15)	*P*-value
Presentation			
BCVA, logMAR (IQR)	0.18 (0-0.70)	0.42 (0.10-1.79)	0.262
RAPD present	36 (24.7%)	5 (35.7%)	0.353
IOP, mmHg (IQR)	19 (15-23)	17 (13-22)	0.455
Ishihara color test 10/10 or 11/11	81 (76.4%)	5 (50.0%)	0.123
EOM restricted	88 (58.7%)	10 (66.7%)	0.595
Follow-up			
BCVA, logMAR (IQR)	0 (0-0.18)	0.10 (0-1.3)	0.130
RAPD present	9 (12.2%)	1 (14.3%)	1.000
IOP, mmHg (IQR)	13 (11-17)	16 (13-18)	0.255
Ishihara color test 10/10 or 11/11	15 (88.2%)	1 (50.0%)	0.298
EOM restricted	17 (21.5%)	3 (42.9%)	0.346

BCVA, best corrected visual acuity; logMAR, log of the minimum angle of resolution; IQR, interquartile range; RAPD, relative afferent pupillary defect; IOP, intraocular pressure; EOM, extraocular movement; The percentages of each category were calculated based on the available data in each category. *p*-values were calculated using Fisher’s exact tests for nominal outcomes and Mann-Whitney U tests for continuous outcomes, comparing patients without cancer and with cancer cohorts.

### Outcomes of orbital or preseptal cellulitis care

In either orbital cellulitis or preseptal cellulitis cohorts, readmission rates, time to follow-up or to readmission were similar between patients with and without cancer (**Table 7**). In patients with cancer, those with orbital cellulitis had a higher overall readmission rate compared to those with preseptal cellulitis (20.0% versus 12.5%, *p* = 1.000).

**Table 7:** Preseptal and orbital cellulitis outcome characteristics.

Preseptal Cellulitis	Patients without Cancer (*N* = 122)	Patients with Cancer (*N* = 8)	*P-value*
Readmission			
In one month	2 (1.6%)	1 (12.5%)	0.175
Overall	7 (5.7%)	1 (12.5%)	0.407
Median time from presentation to			
Follow-up, days (IQR)	81.0 (11.0-437.0)	81.0 (4.0-91.0)	0.229
First readmission, days (IQR)	267.0 (11.0-515.0)	24.0 (24.0-24.0)	0.750
Orbital Cellulitis	Patients without Cancer (*N* = 168)	Patients with Cancer (*N* = 15)	*P*-value
Readmission			
In one month	6 (3.6%)	1 (6.7%)	0.456
Overall	19 (11.3%)	3 (20.0%)	0.397
Median time from presentation to			
Follow-up, days (IQR)	40.5 (13.0-289.5)	172.0 (61.5-441.5)	0.238
First readmission, days (IQR)	74.0 (25.0-199.0)	15.0 (8.0-103.0)	0.300

IQR, interquartile range; *p*-values were calculated using Fisher’s exact tests for nominal outcomes and Mann-Whitney U tests for continuous outcomes for each cellulitis cohort, and within each cohort, calculated between patients without cancer and with cancer.

## Discussion

In this study, patients with previous cancer diagnosis presented with orbital cellulitis and preseptal cellulitis at similar rates (8.2% and 6.2%, *p* = 0.661). This finding is similar to that of the Sagiv and colleagues in a retrospective review of patients with cancer treated for preseptal and orbital cellulitis at a comprehensive cancer center, where 29 of 50 of patients were managed for orbital cellulitis and 21 of 50 with preseptal cellulitis (11). The slightly higher although not significant prevalence of orbital cellulitis over preseptal cellulitis in patients with cancer may be due to their immunosuppressed states, where the immune system response to limiting the breach of orbital infection to the septum is blunted (11). Overall, our study suggests that a prior diagnosis of cancer is not associated with a higher risk of orbital over preseptal cellulitis.

The present study found that patients with cancer presenting with orbital cellulitis were more likely to first present to an ophthalmologist compared to those without cancer (20.0% vs. 7.1%, *p* = 0.111). This trend may reflect the blunted immune response seen in immunocompromised patients, which can lead to less severe or slower onset of symptoms and prompting ophthalmologic clinic visits rather than emergency department presentation (12). However, given orbital cellulitis requires intravenous antibiotics administration and ophthalmologists can be consulted in the setting of an emergency department, the emergency department remains the optimal initial site of care for all patients with orbital cellulitis. Considering case reports of patients presenting with cancer masquerading as orbital cellulitis, such as diffuse large B-cell lymphoma or metastasis from urothelial carcinoma, ophthalmologists can play an additional pivotal role in distinguishing orbital cellulitis from cancer in select cases (13–16).

In addition to bacterial causes, orbital cellulitis can be caused by fungal organisms such as *Mucormycosis* and *Aspergillosis* (3). In this study, patients with cancer were significantly more likely to receive antifungals than those without cancer (46.7% versus 10.1%, *p*=0.001). Although the duration of antifungal treatment was shorter for patients with cancer compared to those without cancer (median 2.0 versus 6.0 days, *p* = 0.395). This pattern may suggest that antifungals in patients with cancer were often started empirically rather than for the treatment of a confirmed fungal infection. In contrast, in the study by Sagiv et al., 9 of the 29 patients (31%) with orbital cellulitis had confirmed fungal causes (11). As Sagiv et al. suggests, patients with cancer who develop orbital cellulitis especially of fungal cause are at a higher risk of death despite their possibly less severe symptoms at presentation (11). It is therefore crucial to consider fungal causes of orbital cellulitis in cancer patients presenting with orbital cellulitis, with broad antimicrobial coverage at admission.

Beyond cancer, other comorbidities can modulate the infection risk and treatment response in orbital cellulitis. In one study of preseptal and orbital cellulitis, diabetes was associated with longer hospital stays for both cellulitis conditions (17). Among patients with orbital cellulitis, those with diabetes also required longer courses of intravenous antibiotics and were more likely to have EOM restriction at follow-up (17). Another study found that adults with hypertension and heart disease had a significantly lower poorer visual acuity recovery after orbital cellulitis management (18). Although our study did not evaluate the role of other co-morbidities beyond cancer on the presentation, management, and outcomes of cellulitis, these studies collectively highlight how pre-existing diagnoses can modulate a patient’s risk profile for successful recovery after orbital cellulitis.

### Limitations

The main limitation of this study is its retrospective nature as orbital and preseptal cellulitis are clinical diagnoses, thus limiting our ability to verify these diagnoses at times with the information included in the electronic medical records (EMR). Due to this limitation, some patients may have been misclassified as we relied on the recorded clinical encounter for the categorization of cohorts. The retrospective nature of the study also led to a small sample size used in the analysis of some variables. Some follow-up and future readmission data may have been missed if patients transitioned care from the Johns Hopkins Hospital system to another institution. Due to these limitations and the patient population being from an academic institution, its conclusions may not be generalizable to all settings in which patients with orbital or preseptal cellulitis present.

### Conclusion

In conclusion, cancer and its treatments may predispose patients to immunosuppression, increasing their risk of infections, including preseptal and orbital cellulitis. Given the urgency of treating orbital cellulitis inpatient compared to the often outpatient-managed preseptal cellulitis, this study aimed to elucidate the course of orbital and preseptal cellulitis in patients with and without cancer using our current management options. When patients with cancer present for preseptal cellulitis, they have similar management and outcome characteristics compared to patients without cancer. Additionally, the present study found that patients with cancer presenting for orbital cellulitis may be started on antifungal treatment at a higher rate compared to patients without cancer, but may be started on steroids later than patients without cancer. Having a high suspicion of orbital cellulitis in patients with cancer, especially hematologic, with broad antifungal coverage may help with prompt orbital cellulitis management.

## Data Availability

The datasets generated during and/or analyzed during the current study are available from the corresponding author on reasonable request. Requests to access the datasets should be directed to Dr. FR, frajaii1@jhmi.edu.
